# Pediatric Ovarian Yolk Sac Tumor Mimicking Appendicitis: A Case Report From a Low‐Resource Setting

**DOI:** 10.1002/ccr3.70013

**Published:** 2024-12-15

**Authors:** William Nkenguye, Alex Mremi, Peter Minja, Jay Lodhia

**Affiliations:** ^1^ Department of Epidemiology and Applied Biostatistics Kilimanjaro Christian Medical University College Moshi Tanzania; ^2^ Kilimanjaro Clinical Research Institute Kilimanjaro Christian Medical Centre Moshi Tanzania; ^3^ Faculty of Medicine Kilimanjaro Christian Medical University College Moshi Tanzania; ^4^ Department of Pathology Kilimanjaro Christian Medical Centre Moshi Tanzania; ^5^ Department of General Surgery Kilimanjaro Christian Medical Centre Moshi Tanzania

**Keywords:** alpha‐fetoprotein, appendicitis, germ cell tumor, ovarian yolk sac tumor, pediatric malignancy

## Abstract

Ovarian yolk sac tumors (OYSTs), also known as endodermal sinus tumors, are rare and highly malignant germ cell tumors, accounting for approximately 1% of all ovarian cancers. They predominantly affect children and young adults, with a rapid growth rate and early metastasis, making early diagnosis and treatment crucial. This report presents the case of a 6‐year‐old female from a low‐resource setting who initially presented with symptoms suggestive of acute appendicitis, including abdominal pain, fever, and vomiting. This was supported by a CT scan; however, intraoperative findings revealed a large right ovarian mass, which was surgically excised via cystectomy and histologically confirmed as an OYST. The patient recovered well postoperatively and was referred for oncological management. This case underscores the importance of considering OYSTs in the differential diagnosis of pediatric abdominal masses and highlights the necessity of a multidisciplinary approach for optimal patient outcomes. Enhanced awareness and improved diagnostic strategies are essential for better management of this rare tumor, particularly in resource‐limited settings.

**Summary**

- Ovarian yolk sac tumors are rare, highly malignant germ cell tumors, representing only 1% of all ovarian tumors.
- Prompt diagnosis and timely management are essential to improve patient outcomes.
- Alpha‐fetoprotein (AFP) serves as a valuable biomarker for both diagnosis and monitoring therapeutic response.
- The standard chemotherapy regimen—bleomycin, etoposide, and cisplatin (BEP)—is widely accepted and has proven effective in managing these tumors.

## 1 Introduction

Ovarian yolk sac tumors (OYSTs), also known as endodermal sinus tumors, are rare and highly malignant germ cell tumors [1, 2]. They account for approximately 1% of all ovarian cancers and are most commonly diagnosed in children and young adults [3, 4]. The incidence of OYSTs is estimated to be about 0.1–0.3 per 100,000 females per year [5]. These tumors are characterized by rapid growth and early metastasis, making timely diagnosis and treatment critical for improving patient outcomes. Alpha‐fetoprotein (AFP) is often elevated in patients with OYSTs, serving as a crucial tumor marker for diagnosis and monitoring response to therapy [6].

In pediatric patients, ovarian yolk sac tumors are a rare but critical differential diagnosis when presenting with acute abdominal pain, which can often mimic more common conditions such as appendicitis. In Africa and other low‐resource settings, the burden of OYSTs, like other pediatric cancers, is not well‐documented because of limited cancer registries and diagnostic facilities [7]. Despite these limitations, germ cell tumors remain a significant portion of pediatric malignancies on the continent [5, 8]. Timely diagnosis and management are further hindered by the lack of specialized diagnostic tools and oncology services [9, 10]. This case highlights the diagnostic difficulty and need for a high index of suspicion when managing pediatric patients in these settings.

## 2 Case Presentation

### 2.1 Case History

A 6‐year‐old female presented to our center with a one‐month history of abdominal pain, initially localized to the right inguinal region and later generalized, accompanied by slight abdominal fullness and intermittent low‐grade fevers. She also experienced postprandial non‐projectile vomiting over the past 2 weeks but denied any weight loss, night sweats, cough, chest pain, abnormal vaginal discharge, abnormal bowel habits, or changes in urinary patterns.

On admission, she was fully conscious and alert, mildly pale, with a respiratory rate of 23 breaths/min, axillary temperature of 39.4°C, blood pressure of 98/67 mmHg, pulse rate of 149 beats per minute, and oxygen saturation of 97% on room air. Her abdomen was slightly distended with symmetrical contours, generalized tenderness, more pronounced in the right iliac region, and reduced bowel sounds. Her cardiovascular and respiratory examinations were normal. Based on the age and clinical presentation, the initial clinical diagnosis was acute appendicitis.

### 2.2 Methods

Laboratory investigations revealed a white blood cell count of 12.07 × 10^9^/L, hemoglobin of 12.6 g/dL, and a platelet count of 440 × 10^9^/L. Serum creatinine was 30 μmol/L, BUN was 2.22 mmol/L, serum potassium was 3.73 mmol/L, and sodium was 134.30 mmol/L. An abdominal ultrasound showed an intra‐abdominal mass of unclear origin. A CT scan of the abdomen and pelvis revealed an appendicular abscess and functional small bowel obstruction. The Alvarado's score was calculated to be 7/10, warranting immediate surgery.

**FIGURE 1:**
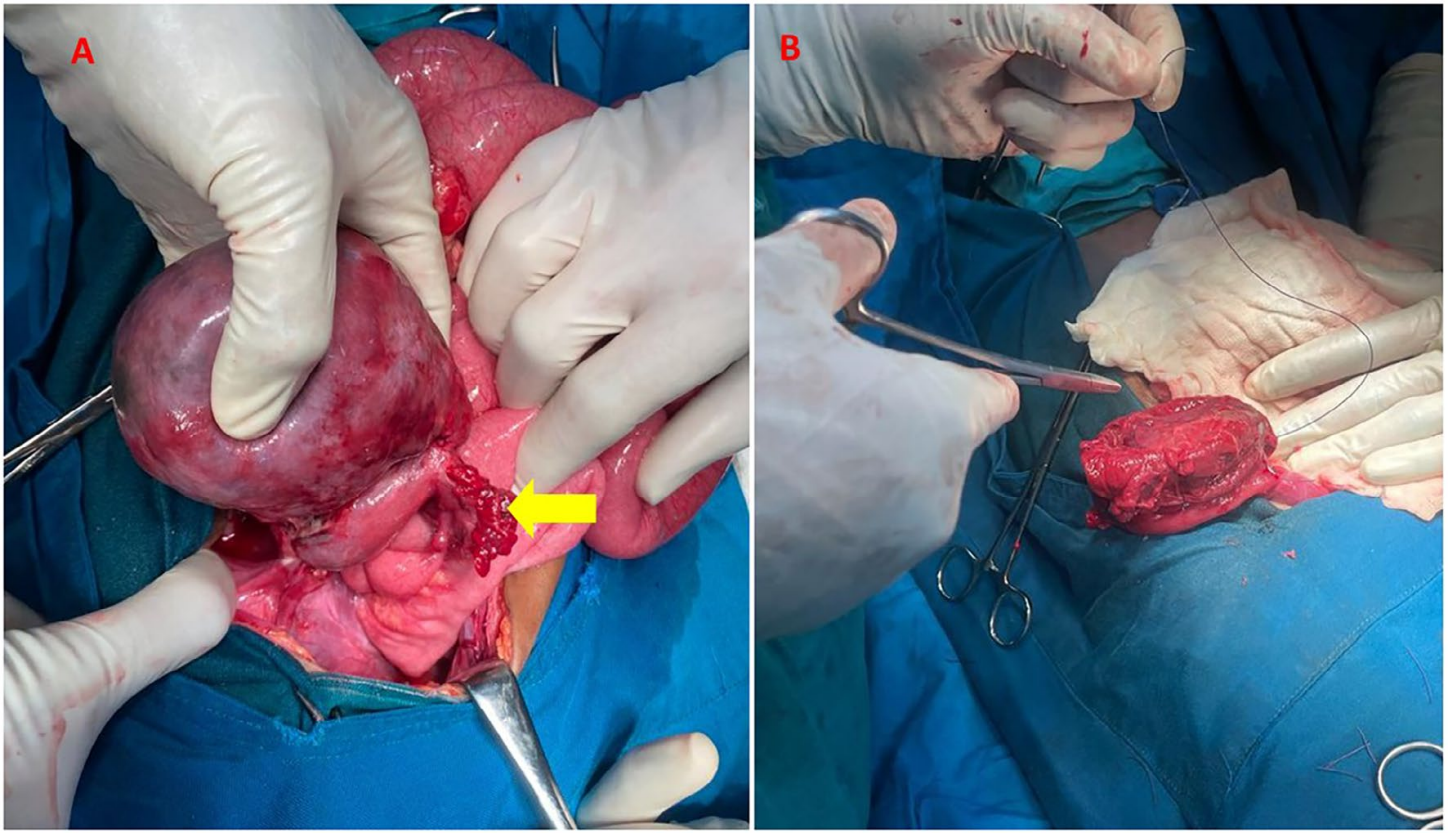
(A) Surgeon holding the ovarian mass attached to the right fallopian tube (yellow arrow showing fimbriae), (B) Post excision of the mass reserving the fallopian tube and ovary.

**FIGURE 2:**
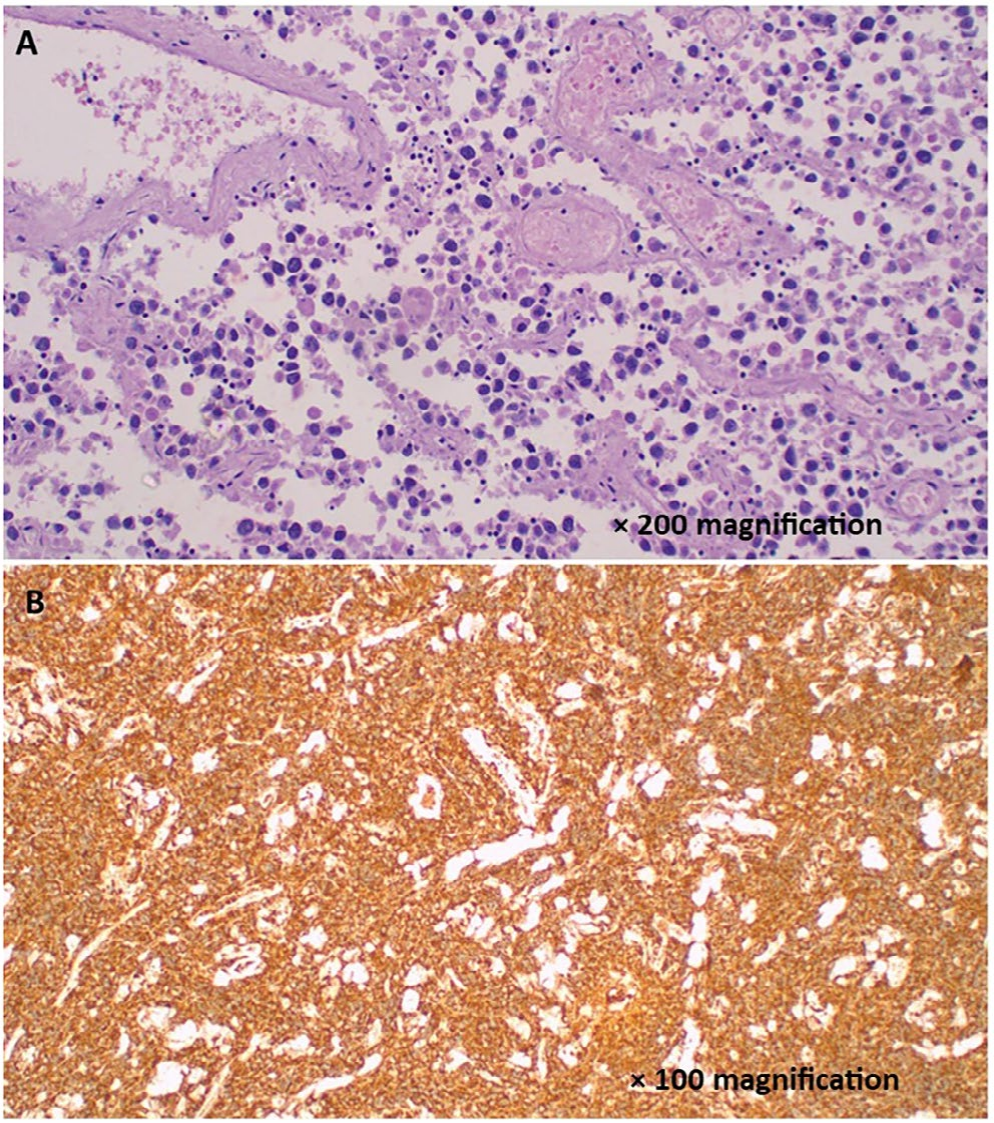
(A) Histopathology of ovarian yolk sac tumor demonstrating microscystic pattern characterized by loose meshwork of anastomosing channels and variably sized cysts; individual tumor cells have a signet ring‐like morphology and the cysts containing eosinophilic hyaline globules and amorphous, eosinophilic acellular basement membrane‐like material; H&E 200 × original magnification. (B) Photomicroscopy of the tumor displaying immunopositivity of the tumor cells with alpha‐fetoprotein (AFP) antibody; IHC 100 × original magnification.

In preparation for laparotomy, the patient was managed with intravenous ceftriaxone, metronidazole, and fluids, kept nil orally, and an NGT was inserted. After counseling the parents and obtaining consent, the child was taken for laparotomy. A lower midline incision was used because of the abscess, allowing better access for complete drainage and exploring the peritoneal cavity. Intraoperative findings however revealed a large (12 by 10 cm) right‐sided ovarian mass with features suggestive of a hemorrhagic ovarian cyst or ovarian tumor (Figure 1). Intraoperative consultation with the gynecology team led to the decision to perform a cystectomy, excising the mass while preserving the ovary and fallopian tube. Histological analysis later confirmed a right ovarian yolk sac tumor (Figure 2).

### 2.3 Conclusion and Results

Postoperatively, the patient's recovery was uneventful and was discharged on Day 5. At her 4‐week follow‐up, her abdominal wound had healed well, and she was referred to the oncology department for further evaluation and management. Her follow‐up CBC was normal with a hemoglobin of 13 g/dL, β‐hCG was 0.43 mIU/mL, α‐fetoprotein was 0.500 IU/mL, and lactate dehydrogenase was 407 U/L. She remains asymptomatic after 6 months and continues 6‐monthly follow‐up to monitor tumor markers.

## 3 Discussion

Ovarian neoplasms are classified by cell origin into three main groups: germ cell, sex cord‐stromal, and epithelial tumors. Prompt differentiation between benign and malignant lesions is essential to reduce morbidity and mortality and to prioritize fertility preservation, especially in pediatric patients. Although most ovarian tumors in pediatric populations are benign, approximately 10%–30% are malignant, with malignancies more common in adolescent girls. Pediatric ovarian tumors represent 1%–3% of all childhood cancers. Diagnosing these tumors is often challenging because of their nonspecific symptoms and varied, subtle imaging findings. Early and accurate diagnosis is key to managing these cases effectively and improving outcomes for pediatric patients [11].

In cases of ovarian tumors, surgeons often face challenging intraoperative decisions, particularly when encountering an unknown pathology. They must determine whether to perform a simple resection, attempt organ‐salvaging techniques, or proceed with staging based on real‐time findings. For many patients, including emergency cases, it is often impractical to conduct a biopsy with frozen sections or obtain a definitive pathological diagnosis during surgery [12]. Among serum tumor markers, AFP is particularly specific for malignant tumors, with others including beta‐human chorionic gonadotropin (β‐hCG), carcinoembryonic antigen (CEA), human placental alkaline phosphatase (hPLAP), and cancer antigen 125 (CA125) providing additional information. However, limitations arise, especially in resource‐limited settings, where these tumor markers may not be available during off‐hours or may take days to return results, hindering timely surgical decision‐making. Moreover, it is important to note that these markers are positive in only about 54% of malignant cases, meaning that a negative result does not conclusively rule out malignancy [13]. These constraints highlight the complexities of intraoperative management and the need for clinical judgment when comprehensive resources are not immediately accessible.

Ovarian yolk sac tumors, though rare, require a high index of suspicion, especially in pediatric patients presenting with abdominal masses and elevated AFP levels [2, 14]. In this case, the initial presentation mimicked appendicitis, which is a common diagnostic pitfall. The systemic inflammatory response caused by the tumor likely explains the fever and tachycardia. Advanced imaging and intraoperative findings were crucial in identifying the true nature of the mass.

Ovarian masses in children and adolescents often present with nonspecific abdominal symptoms, which can easily mimic other conditions, particularly acute appendicitis. Pomeranz et al. highlight that in a series, 59% of cases with ovarian cysts presented with acute abdominal pain, initially suspected to be appendicitis. Differential diagnoses for acute abdominal pain with fever are broad and should include conditions such as diverticulitis (including Meckel's diverticulitis), urolithiasis, enterocolitis, mesenteric adenitis, ovarian torsion, and pelvic inflammatory disease [15, 16] (PID). To accurately diagnose these conditions, various laboratory and imaging studies are valuable. However, clinicians must also consider the limitations of resource availability and the financial implications of extensive testing, especially in resource‐limited settings. A balanced approach to investigation can help in avoiding misdiagnosis while also ensuring efficient and accessible patient care.

This case occurred in a low‐resource setting where advanced diagnostic tools like MRI and preoperative tumor markers are limited. Laparoscopy was not considered due to the lack of specialized equipment and trained surgeon out of hours. Instead, clinical evaluation and basic imaging, such as ultrasound and CT scans, remain vital tools for diagnosis. Given the large size of the mass and its intra‐abdominal location, a lower midline incision was chosen to provide optimal access.

Although a cystectomy was performed to preserve ovarian function, this approach diverges from strict oncology guidelines, which recommend removal of the affected ovary and tube for better staging and prognosis. In resource‐rich settings, comprehensive fertility‐preserving surgical staging is the standard of care. However, in low‐resource settings, such decisions often prioritize immediate clinical outcomes and patient fertility, with follow‐up care coordinated postoperatively.

The patient was referred to the oncology department promptly after surgery for further management, including chemotherapy. Studies have shown that the BEP (bleomycin, etoposide, and cisplatin) regimen is effective in treating OYSTs, significantly improving survival rates [17, 18]. However, late presentation and limited access to comprehensive cancer care remain challenges in low‐resource settings.

To avoid similar misdiagnoses, clinicians should maintain a broad differential diagnosis for pediatric abdominal masses and use available diagnostic tools efficiently [19]. Multidisciplinary collaboration between surgeons, gynecologists, and oncologists is critical for ensuring optimal outcomes.

This case demonstrates the importance of considering ovarian yolk sac tumors in the differential diagnosis of pediatric patients with abdominal masses. Early recognition, appropriate surgical intervention, and histological confirmation are crucial for effective management. The patient's positive outcome underscores the efficacy of a multidisciplinary approach and adherence to standard treatment protocols. Continued follow‐up and chemotherapy are essential to ensure long‐term remission and monitor for recurrence. This case contributes to the body of knowledge on OYSTs and underscores the need for vigilance in diagnosing and managing rare pediatric tumors, particularly in low‐resource settings.

## Author Contributions

**William Nkenguye:** writing – original draft. **Alex Mremi:** formal analysis, resources. **Peter Minja:** data curation, methodology. **Jay Lodhia:** conceptualization, writing – review and editing.

## Ethics Statement

Approval was obtained from the departments of General surgery and the appropriate hospital institutional review board has approved the publication of this case report.

## Consent

Written informed consent was obtained from the child's parents for publication for this case report; additionally, accompanying images have been censored to ensure that the patient cannot be identified. A copy of the consent is available on record.

## Data Availability

The authors have nothing to report.
